# Deep learning based optimal energy management for photovoltaic and battery energy storage integrated home micro-grid system

**DOI:** 10.1038/s41598-022-19147-y

**Published:** 2022-09-07

**Authors:** Md. Morshed Alam, Md. Habibur Rahman, Md. Faisal Ahmed, Mostafa Zaman Chowdhury, Yeong Min Jang

**Affiliations:** 1 Dept. of Electronics Engineering, Kookmin University, Seoul, 02707 South Korea; 2 Dept. of Electrical and Electronic Engineering, Noakhali Science and Technology University, Noakhali 3814, Bangladesh; 3 Dept. of Electrical and Electronic Engineering, Khulna University of Engineering & Technology, Khulna, 9203 Bangladesh

**Keywords:** Electrical and electronic engineering, Energy infrastructure

## Abstract

The development of the advanced metering infrastructure (AMI) and the application of artificial intelligence (AI) enable electrical systems to actively engage in smart grid systems. Smart homes with energy storage systems (ESS) and renewable energy sources (RES)-known as home microgrids-have become a critical enabling technology for the smart grid. This article proposes a new model for the energy management system of a home microgrid integrated with a battery ESS (BESS). The proposed dynamic model integrates a deep learning (DL)-based predictive model, bidirectional long short-term memory (Bi-LSTM), with an optimization algorithm for optimal energy distribution and scheduling of a BESS-by determining the characteristics of distributed resources, BESS properties, and the user’s lifestyle. The aim is to minimize the per-day electricity cost charged by time-of-use (TOU) pricing while considering the day-basis peak demand penalty. The proposed system also considers the operational constraints of renewable resources, the BESS, and electrical appliances. The simulation results from realistic case studies demonstrate the validation and responsibility of the proposed system in reducing a household’s daily electricity cost.

## Introduction

Despite two-way communication facilities and the advanced metering infrastructure (AMI), the optimal management capability of electrical energy among appliances and resources remains behind the rapid growth in power demand. Because of the modern lifestyle of consumers, the application of electrical appliances has increased dramatically. Therefore, it is estimated that global electricity demand will increase by 2.1 per year by 2040 (twice the rate of primary energy demand)^1^. Furthermore , the invention of several renewable energy sources (RES) aims to achieve sustainable electrical energy generation. Photovoltaic (PV) devices are the fastest-growing RES category with a growth rate of 60% whereas the growth rates of wind power and biofuels are 27% and 18%, respectively^2^.

Furthermore, the massive penetration of renewable resources and energy storage systems (ESS) is essential to mitigating electrical energy demand without a higher carbon emission volume. ESS technology can immediately transform and store electrical energy from the electrical power network and inject the electrical energy back according to the applied scheme or when the base units are unavailable for generation^3^. However, integration of ESS with proper management and resource scheduling is arduous. The home energy management system (HEMS)^4^ provides a possible solution by managing the energy consumption and PV generation with the integration of a battery ESS (BESS) that balances supply and demand cost-effectively.

### Related work

Recent studies have evaluated HEMSs from a demand-side management perspective. In considering a real-time electricity price signal, Zhao et al.^5^ proposed a HEMS model for optimal scheduling of domestic appliances. In^6^, day-ahead optimal scheduling for all components was developed by splitting the load and the user’s range anxiety. Although the authors proposed day-ahead scheduling, they did not consider the day-ahead energy consumption and generation profile. Therefore^7^, proposed a HEMS model under user comfort constraints and dynamic pricing to minimize the electricity bill while reducing the daily curtailed energy volume. The study in^8^ developed an integrated solution for dynamically controlling and scheduling the appliances using energy consumption prediction, in which the integration of ESS and prediction of PV generation are absent. In^9^, a rule-based renewable BESS controlling scheme that does not produce the optimal solution and or add demand response flexibility to the appliances was proposed. Reference^10^ studied the charging and discharging of a BESS for power curve smoothing and peak load shaving while integrating the load forecasting method. However, it did not consider the dynamic tariff that would produce the optimal solution over a finite horizon. In^11^, a multi-objective optimization framework for day-ahead scheduling of a microgrid ESS was developed without consideration of the day-ahead generation and consumption constraints.

In a recent study^12^, a two-stage hierarchical HEMS was proposed for effective scheduling of home energy resources depending on the day-ahead and actual operational period. Furthermore, a new HEMS was proposed by scheduling the HERs to minimize the daily electricity cost, considering the demand charge tariff (DCT) and user lifestyle-related operational constraints^13^. In^14^, an optimal day-ahead scheduling approach for shared ESS to reduce energy costs in the smart grid using the agent’s actions was proposed. Xu et al.^15^ proposed a reinforcement learning-based EMS for the home environment but did not consider the scheduling scheme for charging and discharging the BESS. With massive applications of automated appliances, the penetration of PV systems incorporating large-scale ESS with existing systems is imperative to ensure economic and other substantial benefits (e.g., load following, peaking power, and standby reserve).

However, accurate active demand (AD) and PV power generation forecasting are essential for precise scheduling of the BESS in leading continuous and secure power supply by avoiding blackouts. Because of the advancement in deep learning (DL) technology, numerous studies have been conducted on time-series forecasting (i.e., demand, generation, and price) using these techniques. In^16^, the authors used bidirectional long short-term memory (Bi-LSTM) for short-term PV power generation prediction. For ultra-short-term PV power prediction, an improved Bi-LSTM algorithm was proposed to increase performance when the prediction model inputs come from multiple PV output series^17^.

Toubeau et al.^18^ focused on multivariate predictive scenarios for multistep-ahead prediction. The authors in^19^ proposed a Bi-LSTM algorithm for different interval-ahead predictions in large-scale PV power plants. In^20^, a day-ahead peak demand forecasting was accomplished by applying a Bi-LSTM-based sequence to the sequence regression technique. Some recent studies also focused on this algorithm for predicting short-term wind speed^21,22^. However, previous studies^16–20^ only considered the operational constraints of the predictive model for improving performance parameters.

In^23^, the authors propose a HEMS based on binary particle swarm optimization that uses PV power to operate residential appliances and charge/discharge the EV/ESS during low/high tariffs. Similarly, the grey wolf optimization algorithm is designed to schedule charging and discharging periods by considering low/high electricity pricing time in a RES-ESS integrated system^24^. By adjusting energy demand during low/high tariffs, the optimal scheduling of interconnected multi-energy hubs can be achieved, minimizing total operational costs and carbon emissions^25^. Tooryan et al. minimized carbon emissions and increased RES penetration by implementing a PSO algorithm (as a robust meta-heuristic method to schedule BESS) and diesel generators based on the volume of each RES energy generation^26^.

Stochastic optimization for HEMS was developed in the context of electrical energy allocation among the BESS, energy demand, and utility grid, which explicitly integrates probability distributions of trip duration and trip length. The optimization problem was formulated using time-varying electricity pricing and time-varying energy usage^27^. Moreover, a genetic harmony search algorithm was integrated with the home energy management controller to reduce electricity expense and enhance user comfort by considering real-time electricity pricing and critical peak pricing tariffs^28^.

Furthermore, under time-of-use (TOU) pricing and demand-side management, a dynamic programming model is used to govern the power flow flexibly to reduce the net present electricity cost and enhance the self-consumption rate of PV-BESS energy^29,30^. With TOU, a smart energy management system is developed that uses load prediction models for the next 24 h to identify the most appropriate BESS energy management strategy at all time intervals during the day^31^. A mixed-integer linear programming-based robust cost-optimal scheduling algorithm is developed to enhance the overall revenue of a PV-BESS integrated system using RNN and CNN algorithms as a forecasting model^32^. Moreover, model predictive control^33^ and a predictive management strategy^34^ are applied to maximize the self-consumption rate of PV-BESS energy through energy curtailment and scheduling schemes.

Existing studies^6–8,25,28^ focused on shifting the operational time of appliances and RES by considering tariff settings and neglecting the generation and consumption profile. Day and day-ahead scheduling^13,14^ based on single-objective^26^ and multi-objective^11,24^ optimization functions were developed without considering the appliances’ predicted operational restraints. Furthermore^30–34^, did not explore a DL-based day-ahead prediction scheme, whereas the system proposed in this study implements a new, highly accurate prediction model (i.e., Bi-LSTM) for power generation and consumption forecasts. Previous studies considered the power curtailment scheme^33,34^ appliance scheduling scheme^6,30^, and feed-in tariff and TOU scheme^29^, whereas this study uses the constraints drawn from the predicted generation and consumption, real-time state of charge (SoC), and charging and discharging allowance.

From the aforementioned discussion, we may deduce that the existing studies utilized the PV-BESS energy completely on a daily basis owing to not considering the day-ahead constraints. However, if there is no PV generation and no energy stored in the BESS, existing studies fail to determine the optimal strategy for utilizing PV-BESS energy since the system requires power from the grid again, resulting in higher electricity bills due to exceeding the daily grid power allowance. Therefore, the proposed system presents an innovative approach for scheduling and optimization that incorporates day-ahead generation and consumption. The scheduling and optimization procedure was carried out in such a way that the BESS was able to keep energy for mitigating day-ahead energy demand.

### Contributions

This study proposes a novel scheme for a HEMS that optimally schedules and manages the PV-BESS for the customer in a dynamic environment. This study is the first (to the best of our knowledge) to simulate the integration of a predictive model with a control algorithm for optimal scheduling and maximizing the discharge amount by including dynamic tariffs. Significant contributions of this study are as follows:

- In this study, we designed and developed a Bi-LSTM model for day-ahead energy consumption and generation forecasts (described in “Controlling mechanism” section). Training with the predictive model ensures consistent day-ahead forecasting performance by diminishing prediction errors.
- This study proposes a heuristic strategy that considers the impact of predicted energy consumption and generation. The real-time scheduling and optimization technique is modeled in a specific interval by considering the energy demand and generation profile (described in “Simulation study” section).
- The proposed system is evaluated under different scenarios of day-ahead power consumption and PV generation volume by the consumer, whereas existing studies focus only on the present-day scenario. The proposed system’s payback periods are also assessed.

This paper is organized as follows. “System modeling” section describes the modeling of the proposed system. “Controlling mechanism” section presents the control mechanism and operational constraints for the HEMS model. The simulation results and the corresponding discussion are presented in “Simulation study” section, and the conclusion is presented in “Conclusion” section.

## System modeling

**Figure 1:**
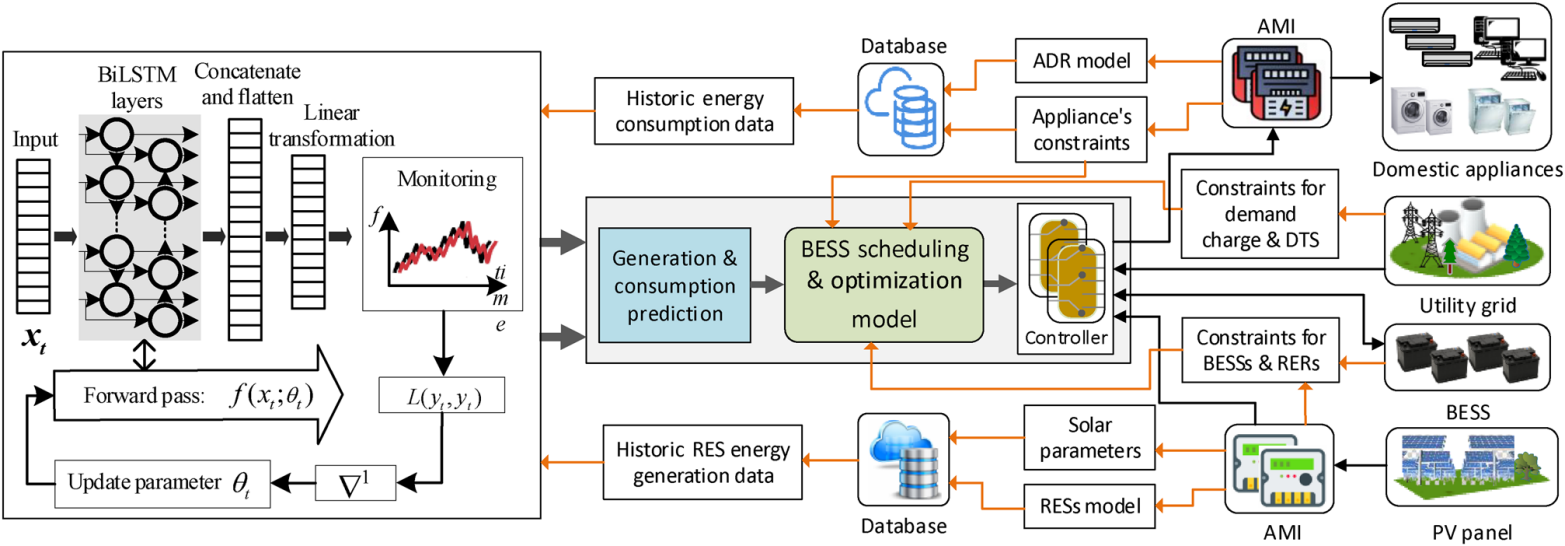
Architecture of the proposed home mirogrid system.

Figure 1 presents the proposed architecture of the home microgrid system. The home is equipped with different appliances, an AMI, and a BESS integrated with PV panels. The BESS is used to store and supply energy based on different constraints. The AMI system is installed, acting as an information provider to the server. The database stores historical data on power generation and consumption used for forecasting. The constraints of the appliances, the BESS, the RES, and the forecasted results are received as inputs in the optimization model at regular intervals. The model also receives the dynamic electricity tariff information at the same intervals. The output of the optimization model provides the scheduling time and amount of power. In this section, the working principle of the Bi-LSTM network and the control algorithm for the ESS are explained.

### Energy consumption and generation forecasting model

**Figure 2:**
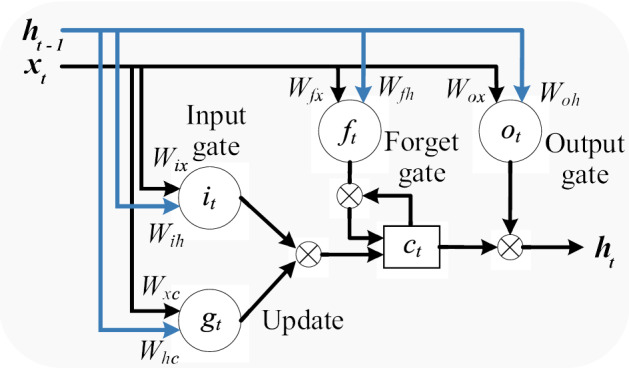
Architecture of LSTM model.

An improved variant of the RNN, known as an LSTM network^35^, removes those limitations by incorporating memory cells and several control gates. Memory cells enable LSTM networks to exploit the long-term dependency of temporal sequences and ensure information propagation through consecutive time steps within internal network structures^36^. Figure 2 presents the LSTM single cell structure consisting of three gates (input gate, output gate, and forget gate). However, an LSTM layer comprises $N_{L}$-connected single cells. Let $X_{t}$ be the measured PV generated power or energy consumption sample at time step *t*. The relationship between the actual and the previously observed data was formulated as follows to predict the 24-h-ahead response of the PV generated power or energy consumption:

1 $$\begin{aligned} ({\hat{Y}_{t+1},\hat{Y}_{t+2},...,\hat{Y}_{t+24}})=LSTM({X}_{t-k+1},...,{X}_{t-1},{X}_{t}) \end{aligned}$$

where $t\in {[k,N-1]}, k$ is the time lag, and *N* is the size of the data. In the equation, $LSTM (\cdot )$ represents the LSTM function of each single cell $L \in [1, N_{L}]$ and is governed by the following:

2 $$\begin{aligned} i_{t} & = \sigma \left( \vec {W}_{i}\left[ \vec {h}_{t-1},x_{t} \right] + b_{i} \right) \end{aligned}$$

3 $$\begin{aligned} f_{t}& = \sigma \left( \vec {W}_{f}\left[ \vec {h}_{t-1},x_{t} \right] + b_{f} \right) \end{aligned}$$

4 $$\begin{aligned} C_{t}& = f_{t}\cdot C_{t-1}+\left( 1-f_{t} \right) \cdot tanh\left( \vec {W}_{c}\left[ \vec {h}_{t-1},x_{t} \right] +b_C \right) \end{aligned}$$

5 $$\begin{aligned} {o_{t}}& = \sigma \left( \vec {W}_{o}\left[ C_{t}, \vec {h}_{t-1},x_{t} \right] +b_o \right) \end{aligned}$$

6 $$\begin{aligned} \vec {h}_{t}& = o_{t}\cdot tanh(C_{t}) \end{aligned}$$

where $i_{t}$, $f_{t}$, and $o_{t}$ are the input gate, forget gate, and output gate. Furthermore, $g_{t}$ is used to update the input signal by modifying the memory state, and $c_{t}$ is the cell state value. However, each gate produces an output based on its individual weight matrix and bias term. Consequently, $h_{t}$ is measured with a concatenating cell state value with the output gate value and i s the cell output value.

**Figure 3:**
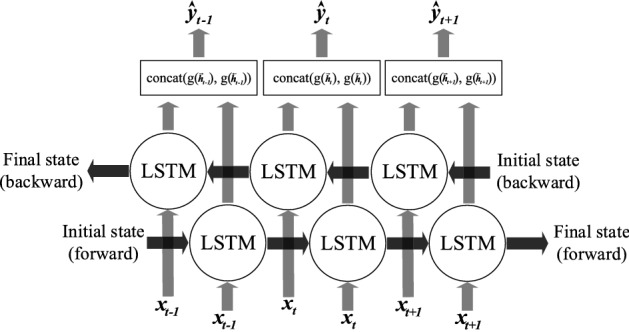
Architecture of Bi-LSTM model.

The sigmoid activation function transforms each gate value into a value between 0 and 1. The cell output finally passes through the hyperbolic tangent activation function (tanh) and predicts $\vec {h}_{t}$. The unidirectional LSTM model processes the input sequence data at each time step *t* using the information contained in the past, ignoring future input-an issue that affects forecasting accuracy in multiple applications. This study adopted a bidirectional learning method that explores both the past (before *t*) and future (after *t*) temporal information among the entire sequence to boost the accuracy of conventional LSTM networks . The principle of this bidirectional learning process exploring both forward and backward sequence directions by two LSTM layers is illustrated in Fig. 3.

The output response at *t* using a hidden vector derived from two LSTM layers is calculated as follows:

7 $$\begin{aligned} {\hat{y}_{t}}=concat(g\left( \overset{{}_{\tiny {\rightarrow }}}{h}_{t} \right) ,g\left( \overset{{}_{\tiny {\leftarrow }}}{h}_{t} \right) ) \end{aligned}$$

The day-ahead power generation and consumption is necessary for scheduling PV-BESS and optimizing the energy charging and discharging allowances. However, the following is a description of the procedure for determining day-ahead power generation and consumption:

- Step 1: At first, zero/nan, and duplicate values from the historic data are removed/replaced through data cleaning process.
- Step 2: The features are selected for the Bi-LSTM model.
- Step 3: The featured data are scaled by applying the data-scaling process.
- Step 4: Initialization of the hyperparameters and designed the Bi-LSTM model.
- Step 5: The data is used to train the Bi-LSTM model and which is saved as a predictive model.
- Step 6: Finally, the real-time data is fed to the predictive model for determining day-ahead energy consumption and PV generation.

### AD scenario

The focus of demand response modeling is cost minimization and user satisfaction. In developing the demand response, the demands of the home microgrid are grouped into different categories depending on the extent to which the demand can be controlled. In this case, the AD of the system is modeled with linear and nonlinear functions according to the properties of the particular user. The dynamic and predicted AD of the household are ${\mathbf {P}}_{AD,t}^{H}(t)$ and ${\mathbf {P}}_{FAD,t}^{H}(t)$. The total power consumption in a household at time $t\in \varvec{\tau }$ can be expressed as follows:

8 $$\begin{aligned} \begin{aligned} {\mathbf {P}}_{AD,t}^{H}(t)&=\sum _{i=1}^{n}P_{d,i}^{ap}(t) \zeta ^{ap}_{i}(t) \end{aligned} \end{aligned}$$

where $\zeta ^{ad}(t)$ is the activity status of the appliances at time *t*. The statuses $\zeta ^{ad}(t)=[0,1]$ and $\zeta ^{ad}(t) \in {\mathbb {Z}}$ vary with continuous changes in demand. For $n\in {\mathbb {N}}$ households in a building, the total power consumption (i.e., predicted) in the period $t\in \varvec{\tau }$ (i.e., on this day) is defined as follows:

9 $$\begin{aligned} {\mathbf {P}}_{FAD}^{B,Total}=\sum _{i=1}^{n}\sum _{j=s}^{e}{\mathbf {P}}_{FAD,\tau }^{H_{i}}(j\tau ) \end{aligned}$$

where $$t=[t_{start},t_{end}]$$, $$t\in {\mathbb {Z}}$$, $$s\in S$$, $$e\in E$$, and $$k\in K$$

$$\begin{aligned} e=\frac{t_{end}}{\tau },\,\,s=\frac{t_{start}}{\tau }\,\text {and}\,\,\,\, k=(\frac{t}{\tau }-1) \end{aligned}$$

10 $$\begin{aligned} t =\left\{ \begin{matrix} t^{h};&{}\text { if { t} is in hour} \\ t^{m};&{}\text {if { t} is in minute}\\ t^{s};&{}\text {if { t} is in second} \end{matrix}\right. \end{aligned}$$

The total predicted power that should be consumed and received by the building from *t* to $t_{end}$ and $t_{start}$ to *t* are as follows:

11 $$\begin{aligned} {\mathbf {P}}_{FAD}^{B,t-t_{end}}=\sum _{i=1}^{n}\sum _{j=1}^{e}{\mathbf {P}}_{FAD,\tau }^{H_{i}}(t+j\tau ) \end{aligned}$$

12 $$\begin{aligned} {\mathbf {P}}_{FAD}^{B,t_{start}-t}=\sum _{i=1}^{n}\sum _{j=1}^{k}{\mathbf {P}}_{FAD,\tau }^{H_{i}}(t-j\tau ) \end{aligned}$$

Because the system has already consumed power from the beginning to time *t*, the actual consumed power and the day-ahead energy consumption of the system are defined as follows:

13 $$\begin{aligned} {\mathbf {P}}_{AD}^{B,t_{start}-t}=\sum _{i=1}^{n}\sum _{j=1}^{k}{\mathbf {P}}_{AD,t}^{H_{i}}(t-j\tau )) \end{aligned}$$

14 $$\begin{aligned} {\mathbf {P}}_{FAD,da}^{B,Total}=\sum _{i=1}^{n}\sum _{j=sa}^{ea}{\mathbf {P}}_{FAD,\tau }^{H_{i}}(j\tau ) \end{aligned}$$

where the time-ahead factors are $a\in {\mathbb {Z}}$, $\left\{ t_{s}, t_{m}, t_{h}\right\} \in ta$ $sa\in SA$, and $ea\in EA$.

15 $$\begin{aligned}&sa=\frac{t_{start}+ta}{\tau }\,\,\text {and} \,\, ea=\frac{t_{end}+a*ta}{\tau },\,\nonumber \\&{\mathbf {P}}_{FAD,d-da}^{B,Total}={\mathbf {P}}_{FAD,da}^{B,Total}+ {\mathbf {P}}_{FAD}^{B,t-t_{end}} \end{aligned}$$

16 $$\begin{aligned}{\mathbf {P}}_{FAD,t}^{B, Total}(t) & =\sum _{i=1}^{n}\sum _{j=1}^{e}{\mathbf {P}}_{FAD,\tau }^{H_{i}}(j\tau )\nonumber \\& \quad -\sum _{i=1}^{n}\sum _{j=1}^{k}{\mathbf {P}}_{AD,t}^{H_{i}}(t-j\tau ) \end{aligned}$$

17 $$\begin{aligned}&{\mathbf {P}}_{FAD,t}^{B,Avg}(t)=\frac{{\mathbf {P}}_{FAD,t}^{B,Total}(t)}{e-k} \end{aligned}$$

18 $$\begin{aligned}{\mathbf {P}}_{FAD,d-da,t}^{B,Total}(t)&=\sum _{i=1}^{n}\sum _{j=1}^{e}{\mathbf {P}}_{FAD,\tau }^{H_{i}}(j\tau )\nonumber \\& \quad +\sum _{i=1}^{n}\sum _{j=sa}^{ea}{\mathbf {P}}_{FAD,\tau }^{H_{i}}(j\tau ) -\sum _{i=1}^{n}\sum _{j=1}^{k}{\mathbf {P}}_{AD,t}^{H_{i}}(t-j\tau )) \end{aligned}$$

19 $$\begin{aligned}&{\mathbf {P}}_{FAD,d-da,t}^{B,Avg}(t)=\frac{{\mathbf {P}}_{FAD,d-da,t}^{B,Total}(t)}{ea-k} \end{aligned}$$

Equations (15) and (16) describe the total predicted demand profile and consumed power. Therefore, the total day and day-ahead forecasted demand are calculated in Eqs. (16) and (18). Equations (17) and (19) define the average day and day-ahead forecasted power demand.

### Distributed generation resource scenario

Consider a residential building with RES, such as a PV power system, where the maximum output power of the PV module is significantly related to its efficiency. In this case, the power generation and the day-ahead power generation are ${\mathbf {E}}_{G,t}^{PV}(t)$ and ${\mathbf {E}}_{FPG,t}^{PV}(t)$. Because the PV power generation depends significantly on the duration of sunlight, the power generated by $n\in {\mathbf {N}}$ number of PV modules at a specific moment is modeled as follows:

20 $$\begin{aligned} \begin{aligned} {\mathbf {E}}_{G,t}^{PV}(t)&=\sum _{i=1}^{n}E_{g,t}^{PV_{i}}(t)\xi ^{PV_{i}}(t) \mathbf {\eta }^{PV_{i}} \end{aligned} \end{aligned}$$

where $\xi ^{PV}(t)$ is the generation status of the PV panel at time *t*. Status $\xi ^{PV}(t)\,\in \,[1,0]$ varies with the continuous response of the PV generation data. The total predicted power generation of the present day is defined as follows:

21 $$\begin{aligned}&{\mathbf {E}}_{FPG,Total}^{PV}=\sum _{i=1}^{n}\sum _{j=0}^{e_{PV}\in E}{\mathbf {E}}_{FPG,\tau }^{PV_{i}}(t_{st}+j\tau ) \end{aligned}$$

22 $$\begin{aligned}&{\mathbf {E}}_{FPG,t_{start}-t}^{PV}=\sum _{i=1}^{n}\sum _{j=1}^{k_{PV}\in K}{\mathbf {E}}_{FPG,\tau }^{PV_{i}}(t-j\tau ) \end{aligned}$$

23 $$\begin{aligned}&{\mathbf {E}}_{G,t_{start}-t}^{PV}=\sum _{i=1}^{n}\sum _{j=1}^{k_{PV}\in K}{\mathbf {E}}_{G,\tau }^{PV_{i}}(t-j\tau ) \end{aligned}$$

The total predicted power and actual generated power are calculated in Eqs. (22) and (23). The total day-ahead energy generation profile is the summation of generation at time *t* to the day-ahead generation end time and is expressed as follows:

24 $$\begin{aligned} {\mathbf {E}}_{FPG,d-da}^{PV,Total}=\sum _{i=1}^{n}\sum _{j=sa_{PV}}^{ea_{PV}}{\mathbf {E}}_{PG,\tau }^{PV_{i}}(j\tau ) + \sum _{i=1}^{n}\sum _{j=1}^{e_{PV}\in E}{\mathbf {E}}_{FPG,\tau }^{PV_{i}}(t+j\tau ) \end{aligned}$$

where $sa_{PV}\in SA$ and $ea_{PV}\in EA$. The predicted generated power at *t* to $t_{end}$ and the average predicted generated power at that time are calculated as follows:

25 $$\begin{aligned} {\mathbf {E}}_{FPG,T}^{PV}(t)=\sum _{i=1}^{n}\sum _{j=1}^{e_{PV}}{\mathbf {E}}_{FPG,\tau }^{PV_{i}}(j\tau )-\sum _{i=1}^{n}\sum _{j=1}^{k_{PV}}{\mathbf {E}}_{FPG,t}^{PV_{i}}(t-j\tau ) \end{aligned}$$

26 $$\begin{aligned} {\mathbf {E}}_{FPG,Avg}^{PV}(t)=\frac{{\mathbf {E}}_{FPG,T}^{PV}(t)}{e_{PV}-k_{PV}} \end{aligned}$$

27 $$\begin{aligned} \begin{aligned} {\mathbf {E}}_{FPG,d-da,t}^{PV}(t)&=\sum _{i=1}^{n}\sum _{j=1}^{e_{PV}}{\mathbf {E}}_{FPG,\tau }^{PV_{i}}(j\tau )+\sum _{i=1}^{n}\sum _{j=sa_{PV}}^{ea_{PV}}{\mathbf {E}}_{FPG,\tau }^{PV_{i}}(j\tau ) \\ & \quad -\sum _{i=1}^{n}\sum _{j=1}^{k_{PV}}{\mathbf {P}}_{G,t}^{H_{i}}(t-j\tau )) \end{aligned} \end{aligned}$$

28 $$\begin{aligned} {\mathbf {E}}_{FPG,d-da,Avg}^{PV}(t)=\frac{{\mathbf {E}}_{FPG,d-da,t}^{PV}(t)}{ea_{PV}-k_{PV}} \end{aligned}$$

The day-ahead predicted generated power and their average amount are calculated in Eqs. (26) and (28).

### BESS scenario

The objective function for optimal energy management and scheduling in the BESS integrated system aims to maximize reliability and minimize the energy cost of the user. The proposed system focuses on several HERs connected in a building. The operational constraints of ESS in different stages of the optimization formulation are calculated by Eqs. (29) and (30). Consider that $n\in N$ number of BESS units are deployed in the system. The instant volume of storage and initial energy can be determined as follows:

29 $$\begin{aligned} \begin{aligned} {\mathbf {P}}_{S,T}^{BESS}(t)&= \sum _{id=1}^{n}P_{C,id}^{BESS}\xi _{t,id} ^{BESS}(t) {SOC}_{t,id}^{BESS} \end{aligned} \mathbf {SOC}_{C,t}^{BESS} = \left\{ \mathbf {SOC}_{min,id}^{BESS},\mathbf {SOC}_{max,id}^{BESS} \right\} \nonumber \\ \end{aligned}$$

30 $$\begin{aligned} {\mathbf {P}}_{T,IE}^{BESS}(t)=\sum _{id=1}^{n}P_{C,id}^{BESS}{SOC}_{t,id}^{BESS}(t-1) \end{aligned}$$

where $\xi ^{PV}(t)\,\in \,[1,0]$ and ${SoC}_{t,id}^{BESS} \in \mathbf {SoC}_{t,id}^{BESS}$. The amount of energy that can be supplied to the system and stored in the BESS at time *t* can be defined as follows:

31 $$\begin{aligned} \begin{aligned} {\mathbf {P}}_{T,DA}^{BESS} (t)&=\sum _{id=1}^{n}P_{C,id}^{BESS}(t)({SOC}_{t,id}^{BESS}(t) -SOC_{min,id}^{BESS}), \\ {}&SOC_{min,id}^{BESS} \in \mathbf {SOC}_{min,id}^{BESS} \end{aligned} \end{aligned}$$

32 $$\begin{aligned} \begin{aligned} {\mathbf {P}}_{T,CA}^{BESS} (t)&=\sum _{id=1}^{n}P_{C,id}^{BESS}(t)(SOC_{max,id}^{BESS}-{SOC}_{t,id}^{BESS}(t)), \\ {}&SOC_{max,id}^{BESS} \in \mathbf {SOC}_{max,id}^{BESS} \end{aligned} \end{aligned}$$

33 $$\begin{aligned} {SOC}_{t,id}^{BESS}(t) < SOC_{min,id}^{BESS},\,\, S_{t, c}^{BESS}(t)\in {\mathbf {S}}_{t, c}^{BESS} \end{aligned}$$

Therefore, constraints for the beginning of charging (such as a particular level of generation) are considered. The set of this threshold level of PV panel generation is defined as ${\mathbf {E}}_{G,th}^{PV}$. If multiple panels connected with a single ESS are considered, then the sum of the threshold level is defined as follows:

34 $$\begin{aligned} {\mathbf {E}}_{G,T,th}^{PV} = \sum _{i=1}^{n} E_{G,th}^{PV_i}, \ E_{G,th}^{PV}\in {\mathbf {E}}_{G,th}^{PV} \end{aligned}$$

35 $$\begin{aligned} {\mathbf {E}}_{G,T,th}^{PV}\leqslant {\mathbf {E}}_{G,t}^{PV}(t),\,\, S_{t, c}^{BESS} \end{aligned}$$

36 $$\begin{aligned} {SOC}_{t,id}^{BESS}(t)> SOC_{max,id}^{BESS},\,\, (1-S_{t, c}^{BESS}) \end{aligned}$$

37 $$\begin{aligned} SOC_{t+1,id}^{BESS}(t+1)= \frac{\left( {\mathbf {E}}_{G,t}^{PV}(t)-{\mathbf {P}}_{AD,t}^{B}(t)\right) }{ P_{C,id}^{BESS}} +SOC_{t,id}^{BESS}(t) \end{aligned}$$

38 $$\begin{aligned} {\mathbf {P}}_{AD,t}^{B}(t)=P_{t,d}^{grid}(t) \end{aligned}$$

where $S_{t, c}^{ESS}$ are binary variables expressing the charging and discharging status of the ESS. Because the charging and discharging process will not occur simultaneously, the appliance will draw power from the grid in the charging period as calculated in Eq. (38).

### Electricity price modeling

The per-unit prices of electricity and DCT depend on the volume of the contracted load, the level of the supply voltage, the types of consumers, and their locations. Because this study focuses on residential consumers, demand charge and energy charge are considered. From this perspective, the per-unit cost is determined based on the market price of an electricity company^37^. The constraints for the DCT can be expressed as follows:

39 $$\begin{aligned} T_{t,DCT}^{Th}= \left\{ \begin{matrix} T_{t,DCT}^{th_{0}}, \,\,\,\text {if}\,\,\,\,P^{th_0}_{C}< P^{B,total}_{C}\le P^{th_1}_{C}\\ T_{t,DCT}^{th_{1}}, \,\,\,\text {if} \,\,\,\, P^{th_1}_{C}< P^{B,total}_{C}\le P^{th_2}_{C}\\ \vdots \\ T_{t,DCT}^{th_{n-1}}, \,\,\,\text {if} \,\, \,\,P^{th_{n-1}}_{C} < P^{B,total}_{C}\le P^{th_n}_{C} \end{matrix}\right. \end{aligned}$$

**Figure 4:**
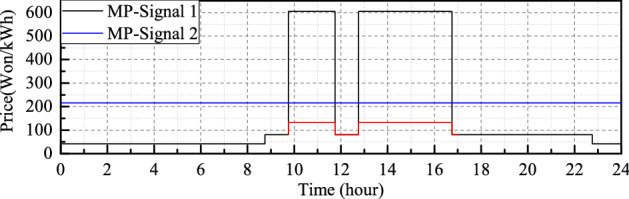
Daily market price (MP) signal (hourly).

Therefore, a fixed TOU tariff is considered^38^, in which electricity is priced in three different fixed intervals (i.e., off-peak, medium peak, and peak) in a day, including weekdays and weekends, as depicted in Fig. 4. The hourly stepped price signal is defined as follows:

40 $$\begin{aligned} T_{t,TOU}^{tf} (t)= \left\{ \begin{matrix} T_{t,TOU}^{op}, \,\,\,\text {if}\,\,\,\,t_{op}^{srt} \le t< t_{op}^{end}\\ T_{t,TOU}^{mp}, \,\,\,\text {if} \,\,\,\, t_{mp}^{srt} \le t < t_{mp}^{end}\\ T_{t,TOU}^{p}, \,\,\,\text {if} \,\, \,\,t_{p}^{srt} \le t\le t_{p}^{end} \end{matrix}\right. \end{aligned}$$

where $T_{t,TOU}^{op}$, $T_{t,TOU}^{mp}$, and $T_{t,TOU}^{p}$ are the tariff amounts in the off-peak, medium peak, and peak times.

## Controlling mechanism

The models of energy demand, distributed generation resources (i.e., PV), and BESS are designed with different control approaches to reduce the demanded energy from the grid and use the generated power appropriately. The constraints of a BESS and dynamic pricing system are also formulated to ensure lower electricity costs. In this study’s proposed model, it is assumed that consumers draw energy from the main grid. Furthermore, the DG is deployed to the systems with an integrated BESS. The control approaches are classified by considering their given constraints. After each iteration, a specific constraint-handling mechanism is applied to each control mode. Therefore, in the evaluation process of the proposed algorithm, after each iteration, the constraints for scheduling and discharging are updated. The fundamental condition for supplying power is defined as follows:

41 $$\begin{aligned} {\mathbf {E}}_{G,T,th}^{PV}> {\mathbf {E}}_{G,t}^{PV}(t),\,\, (1-S_{t, c}^{BESS} ) \end{aligned}$$

42 $$\begin{aligned} SOC_{min,id}^{BESS}\leqslant SOC_{t,id}^{BESS}(t)\leqslant SOC_{max,id}^{BESS} \end{aligned}$$

If the PV generation and the SoC range constraints of the BESS are violated in the given charging period, the charging and discharging schedules of the BESS in that time will be adjusted with updated boundary values. We focused on three control modes for an optimal solution by considering different constraints, as follows.

### Primary control mode (PCM)

The primary control mode (PCM) is a fundamental control approach for scheduling the BESS where the supplying power is controlled under specific constraints (time function). It provides minimum discharging allowance varies with forecasted average demand within limited constraints as follows:

43 $$\begin{aligned} {\mathbf {P}}_{AD,t}^{B}(t) \geqslant {\mathbf {P}}_{FAD,t}^{B,Avg}(t),\,\, {\mathbf {S}}_{PCM,Dis}^{BESS}(t) \end{aligned}$$

44 $$\begin{aligned} \begin{aligned} {\mathbf {P}}_{PCM,Dis}^{BESS}(t)= {\mathbf {P}}_{AD,t}^{B}(t)- {\mathbf {P}}_{FAD,t}^{B,Avg}(t) \end{aligned} \end{aligned}$$

45 $$\begin{aligned} {\mathbf {P}}_{AD,t}^{B}(t) < {\mathbf {P}}_{FAD,t}^{B,Avg}(t),\,\, (1-{\mathbf {S}}_{PCM,Dis}^{BESS}(t)) \end{aligned}$$

46 $$\begin{aligned} \begin{aligned} {\mathbf {P}}_{PCM,Dis}^{BESS}(t)= 0 \end{aligned} \end{aligned}$$

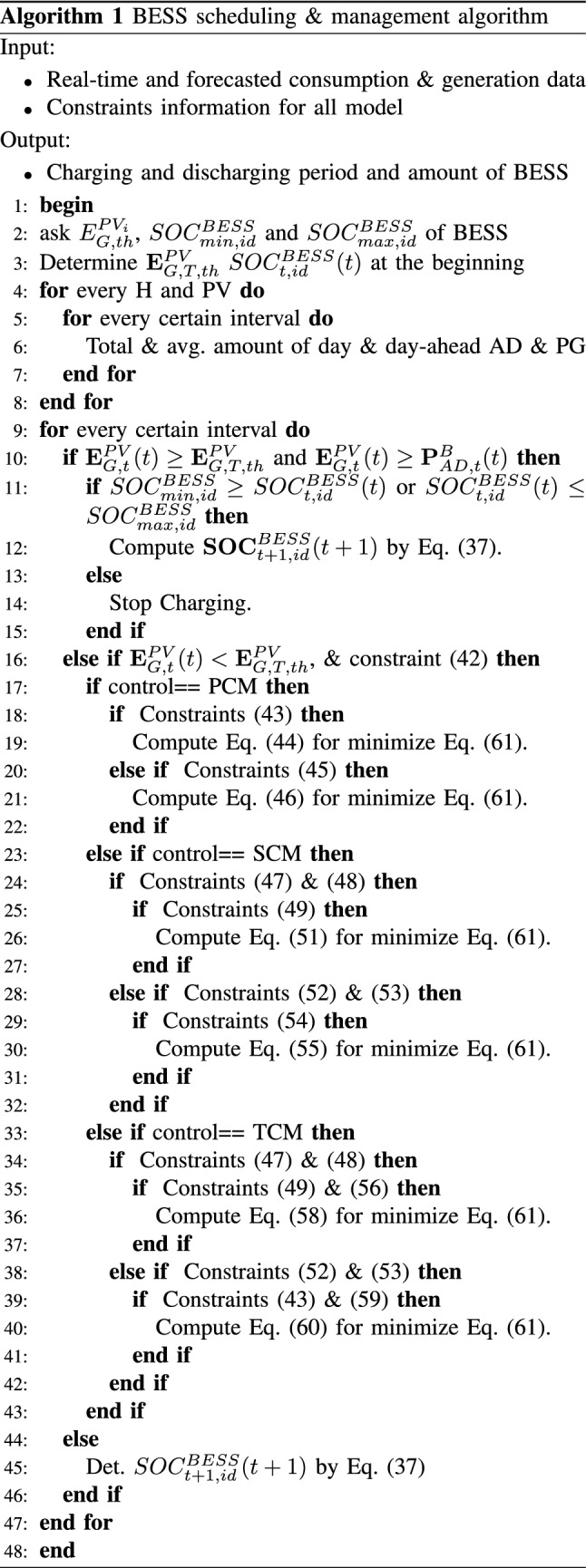


### Secondary control mode (SCM)

In these cases, the objective function is modified by adding a term associated with the day-ahead power generation and current generation volume. Therefore, a new factor used for controlling discharge amount is integrated into the objective function to reduce energy cost by considering the day-ahead power consumption and generation profile. The constraints and required function of the secondary control mode (SCM) are as follows:

47 $$\begin{aligned} \begin{aligned} {\mathbf {P}}_{FAD,t}^{B,Avg}(t)\geqslant {\mathbf {P}}_{FAD,d-da,t}^{B,Avg}(t) \end{aligned} \end{aligned}$$

48 $$\begin{aligned} \begin{aligned} {\mathbf {E}}_{FPG,d-da,Avg}^{PV}(t) \geqslant {\mathbf {E}}_{PG,Avg}^{PV}(t) \end{aligned} \end{aligned}$$

49 $$\begin{aligned} {\mathbf {P}}_{FAD,d-da,t}^{B,Avg}(t)\leqslant {\mathbf {P}}_{AD,t}^{B}(t),\,\, {\mathbf {S}}_{SCM,Dis}^{BESS}(t) \end{aligned}$$

50 $$\begin{aligned} F_{Dis,t}^{SCM}(t) = \frac{{\mathbf {E}}_{FPG,Avg}^{PV}(t)}{{\mathbf {E}}_{FPG,d-da,Avg}^{PV}(t)} \end{aligned}$$

51 $$\begin{aligned} \begin{aligned} {\mathbf {P}}_{SCM,Dis}^{BESS}(t)= {\mathbf {P}}_{AD,t}^{B}(t)- {\mathbf {P}}_{FAD,t}^{B,Avg}(t)*F_{Dis,t}^{SCM}(t) \end{aligned} \end{aligned}$$

where $F_{Dis,t}^{SCM}$ is the secondary discharging factor. If the system’s AD is higher than the average day-ahead power prediction, the volume of energy supply to the system will differ. The constraints for this particular period are:

52 $$\begin{aligned} \begin{aligned} {\mathbf {P}}_{FAD,t}^{B,Avg}(t)< {\mathbf {P}}_{FAD,d-da,t}^{B,Avg}(t) \end{aligned} \end{aligned}$$

53 $$\begin{aligned} \begin{aligned} {\mathbf {E}}_{FPG,d-da,Avg}^{PV}(t) < {\mathbf {E}}_{FPG,Avg}^{PV}(t) \end{aligned} \end{aligned}$$

54 $$\begin{aligned} {\mathbf {P}}_{FAD,t}^{B,Avg}(t)\leqslant {\mathbf {P}}_{AD,t}^{B}(t),\,\, {\mathbf {S}}_{SCM,Dis}^{BESS}(t) \end{aligned}$$

55 $$\begin{aligned} \begin{aligned} {\mathbf {P}}_{SCM,Dis}^{BESS}(t)= {\mathbf {P}}_{AD,t}^{B}(t)- \frac{{\mathbf {P}}_{FAD,t}^{B,Avg}(t)}{F_{Dis,t}^{SCM}(t)} \end{aligned} \end{aligned}$$

### Tertiary control mode (TCM)

In this case, the additional benefits of the systems are encouraged based on the tariff plan. The discharging process of the BESS will be designed in accordance with the energy consumption, generation, and dynamic pricing of electricity cost. The main feature of this optimization mode is to provide a higher amount of energy to the load during periods of higher tariffs.

56 $$\begin{aligned} \begin{aligned} {T}_{t,TOU}^{tf}(t)\geqslant {T}_{t,TOU}^{op}(t),\,\, {\mathbf {S}}_{TCM,Dis}^{BESS}(t) \end{aligned} \end{aligned}$$

57 $$\begin{aligned} T_{Dis,Fac,t}^{TCM}(t) = \frac{T_{Max,t}^{TCM}}{T_{ta,t}^{TCM}(t)} \end{aligned}$$

58 $$\begin{aligned} \begin{aligned} {\mathbf {P}}_{TCM,Dis}^{BESS}(t)= {\mathbf {P}}_{AD,t}^{B}(t)- {\mathbf {P}}_{FAD,t}^{B,Avg}(t)\frac{F_{Dis,t}^{SCM}(t)}{T_{Dis,Fac,t}^{TCM}(t)} \end{aligned} \end{aligned}$$

59 $$\begin{aligned} \begin{aligned} {T}_{t,TOU}^{mp}(t) \leqslant {T}_{t,TOU}^{tf}(t)\leqslant {T}_{t,TOU}^{p}(t),\,\, {\mathbf {S}}_{TCM,Dis}^{BESS}(t) \end{aligned} \end{aligned}$$

60 $$\begin{aligned} \begin{aligned} {\mathbf {P}}_{TCM,Dis}^{BESS}(t)= {\mathbf {P}}_{AD,t}^{B}(t)- {\mathbf {P}}_{FAD,t}^{B,Avg}(t)\frac{F_{Dis,t}^{SCM}(t)}{T_{Dis,Fac,t}^{TCM}(t)} \end{aligned} \end{aligned}$$

where $T_{Dis,Fac,t}^{TCM}$ is the tertiary discharging factor. The objective function for three control mode is formulated as follows:

61 $$\begin{aligned} \begin{aligned} min\sum _{t=t_{start}}^{t_{end}}\left( \mathbf {SOC}_{t,id}^{BESS}(t) - \frac{{\mathbf {P}}_{CM,Dis}^{BESS}(t)}{P_{C,id}^{BESS}}\right) \end{aligned}\nonumber \\ {\mathbf {P}}_{CM,Dis}^{BESS}(t) = \left\{ {\mathbf {P}}_{PCM,Dis}^{BESS}(t),{\mathbf {P}}_{SCM,Dis}^{BESS}(t),{\mathbf {P}}_{TCM,Dis}^{BESS}(t) \right\} \nonumber \\ {\mathbf {S}}_{CM,Dis}^{BESS}(t) = \left\{ {\mathbf {S}}_{PCM,Dis}^{BESS}(t),{\mathbf {S}}_{SCM,Dis}^{BESS}(t),{\mathbf {S}}_{TCM,Dis}^{BESS}(t) \right\} \end{aligned}$$

The step-by-step process of the proposed optimization algorithm is described in Algorithm 1.

## Simulation study

The real-time energy demand data of the multiple-unit building has been measured by a smart meter, and the PV generation data has been measured from the PV site. The duration of historical data was more than 6 months. The scheduling process starts at 12:00 a.m., continues until 12:00 p.m., and consists of 96 time intervals (every 15 min). The dynamic tariff, including peak, medium peak, and non-peak, are presented in Fig. 4. The proposed forecasting algorithm is implemented in the Python platform. The integration of one-day-ahead energy consumption and the PV generation and power management algorithm is simulated on an HP Z8 G4 Workstation with 256 GB of memory and Intel $\circledR$ X$\circledR$(R) Gold 5222 CPU @ 3.80GHz 3.79 GHz processors.

The key performance indicators of the forecasting algorithm for multi-step day-ahead prediction and PV power generation are presented in Tables 1 and 2. However, we observed a weekly repeating pattern after analyzing the power consumption profile. Therefore, the predictive model has been trained based on 672 previous time-steps data to predict 96 steps ahead of the power consumption data. In Table 1, the performance in terms of the mean absolute percentage error (MAPE) is 13.69% for $H_{3}$, which is the lowest value among the households. The mean squared error (MSE) and the mean absolute error (MAE) values for $H_{1}$ are less than those for the other households (.017 and .029 kW).

In contrast, the forecasting of day-ahead PV power generation is performed by measuring 96 time-steps of back power generation data. The performance of the forecasting model in terms of MAPE for $H_{4}$ is higher than the other households (MAPE = $16.421\%$). The lowest MSE value is from $H_{2}$ and $H_{3}$ (.0001 kW). Furthermore, the MAE value of $H_{1}$ is very lower when compared with the others. Table 3 presents the total day and day-ahead demand, PV generation, and energy from the grid (i.e., GS) for the entire day. The BESS capacity (Cap) and initial energy (IE) are also presented.

**Table 1:** Performance of the day-ahead forecasting model.

Performance indicators	$H_{1}$	$H_{2}$	$H_{3}$	$H_{4}$
MSE (kW)	0.017	0.027	0.021	0.027
MAE (kW)	0.029	0.047	0.037	0.042
MAPE	20.54%	20.42%	13.69%	15.12%

**Table 2:** Performance of the day-ahead forecasting model.

Performance indicators	$H_{1}$	$H_{2}$	$H_{3}$	$H_{4}$
MSE (kW)	0.0005	0.0001	0.0001	0.0002
MAE (kW)	0.0122	0.0061	0.0061	0.0071
MAPE	17.953%	21.784%	19.935%	16.421%

**Table 3:** Parameters for different households in [kWh].

	AD	FAD	PVG	FPVG	PVS	GS	Cap	IE
$H_{1}$	15.03	12.50	12.79	8.80	6.81	8.22	8.00	1.75
$H_{2}$	18.92	16.80	12.70	11.94	6.26	12.66	9.50	3.00
$H_{3}$	18.89	18.23	11.61	14.10	5.0	13.87	9.00	3.75
$H_{4}$	16.51	16.29	12.54	12.70	5.11	11.39	7.00	1.50

The daily electricity cost with and without a PV system is presented in Table 4. Because the primary goal is to manage electricity costs in a house by adapting the algorithm to users, three controlling modes are implemented, followed by the user’s energy consumption, generation, and BESS status. The PCM enables the management systems to control the BESS when the AD crosses the average demand. The SCM and TCM modes are designed by considering the day-ahead generation and consumption level.

**Table 4:** Daily electricity cost of households.

House no.	$H_{1}$	$H_{2}$	$H_{3}$	$H_{4}$
Cost [KRW]	1226.59	1410.72	1483.18	1346.46
Cost with PV [KRW]	501.55	744.97	775.51	545.72

Therefore, the tertiary control mode (TCM) enables the BESS to discharge energy by associating it with the time-varying tariff plan proposed by the power company. The scheduling period of the ESS is predetermined for all three scenarios. Renewable power generation (e.g., PV, wind, and tidal) is strongly dependent on meteorological data. Thus, the probability of changing the power generation volume is high. Given these phenomena, four cases are considered for applying our algorithm:

- Scenario 1 ($S_{1}$): When the present volume of consumption and generation are higher than day-ahead consumption and generation volume.
- Scenario 2 ($S_{2}$): When the present demand exceeds the day-ahead demand, yet the day-ahead generation is approximately equal to the current generation.
- Scenario 3 ($S_{3}$): When the day-ahead generation exceeds the current generation, yet the current demand is approximately equal to the day-ahead demand.
- Scenario 4 ($S_{4}$): When the volume of day-ahead generation and demand are approximately equal to the current generation and demand.

**Figure 5:**
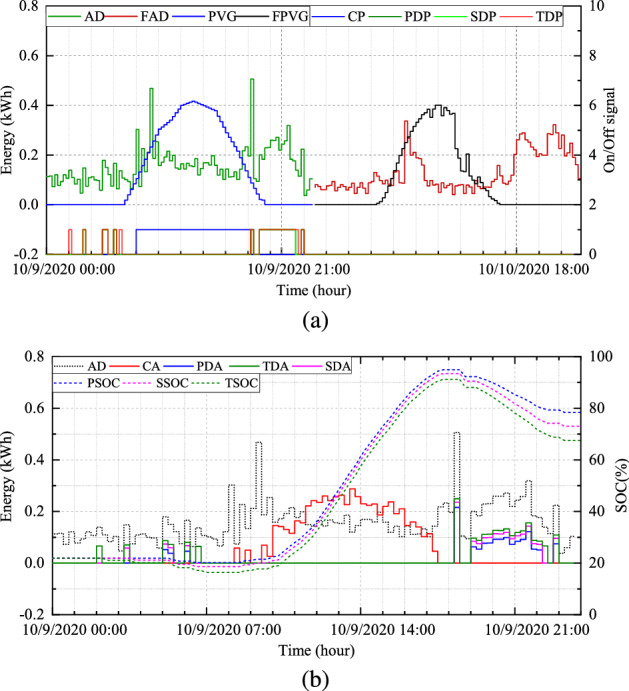
(**a**) Day-ahead results and scheduling periods and (**b**) charging, discharging, and SOC at normal condition for $H_{1}$.

In assessing the robustness of the proposed system, we selected $H_{1}$ for $S_{1}$, $H_{2}$ for $S_{2}$, $H_{3}$ for $S_{3}$, and $H_{4}$ for $S_{4}$. Figure 5a illustrates the simulation results of BESS scheduling in terms of energy (kW) against time (hour) for $H_{1}$. The other ordinary axis corresponds to the turn-on and turn-off signals of the deployed BESS. The lower portion represents the charging period and discharging period of each mode, such as the primary discharging period (PDP), the secondary discharging period (SDP), and the tertiary discharging period (TDP). T he TCM always provides maximum discharging allowance for the BESS. The day-ahead forecasted PV power generation (PVG) and forecasted AD (FAD) are also presented.

**Figure 6:**
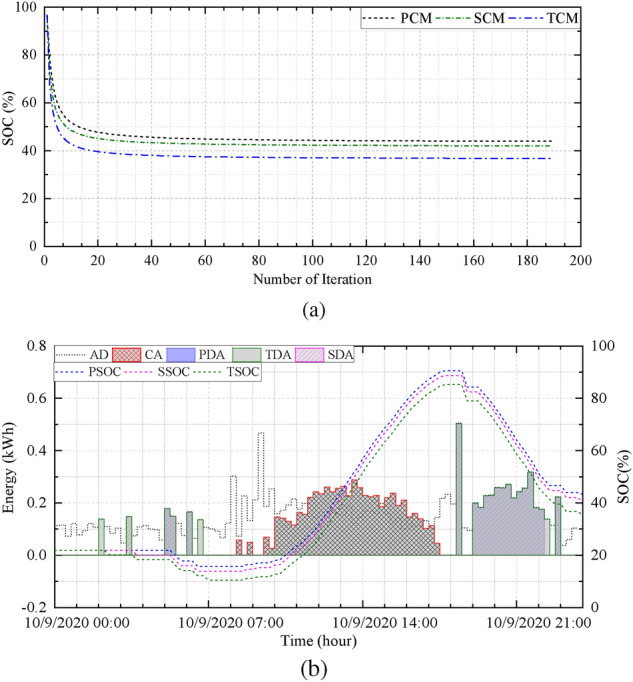
(**a**) Convergence curve, and (**b**) charging, discharging, and SOC at optimal condition for $H_{1}$.

Figure 5b depicts the energy demand, charging and discharging amount, and SoC of BESS in three modes under normal conditions. The discharging amounts of the three different modes are expressed as the primary discharge amount (PDA), the secondary discharge amount (SDA), and the tertiary discharge amount (TDA). Similarly, Fig. 6a illustrates simulation results under optimal conditions, and Fig.6b presents the convergence curve of the proposed optimization model.

Because the motive of the proposed system is to adjust the scheduling period and the discharge amount of the BESS with the assistance of day-ahead PV power generation and consumption, the proposed optimization model is capable of accomplishing the assignment. As depicted in Fig. 5b, the SoC of the BESS is relatively high such as in the end of the period, despite having a discharge allowance. The final SoC under normal conditions in different modes are PCM-78.43%, SCM-73.00%, and TCM-67.44% while optimal conditions achieve PCM-43.91%, SCM-41.98%, and TCM-36.76%.

**Figure 7:**
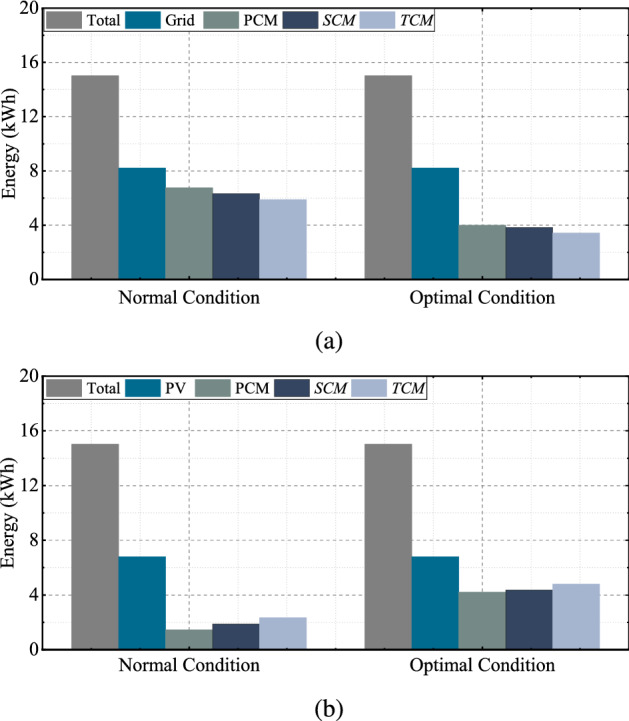
(**a**) Grid energy supply and (**b**) BESS energy supply at normal and optimal condition for $H_{1}$.

After comparing the results, optimal conditions provide a higher discharging allowance within the given constraints. The BESS retains a portion of energy instead of complete discharging caused by insufficient day-ahead PV generation according to the $S_{1}$. Figure 7a illustrates the amount of energy supplied by the grid required to meet the electricity demand at normal and optimal conditions. Similarly, Fig.7b illustrates the volume of BESS energy used by the household. The primary and TCM provides minimum and maximum energy discharging allowance of BESS. These results suggest that the application of the proposed scheme can reduce the supply of grid energy and increase the usage of renewable energy by providing proper coordination among the grid, PV, and BESS system.

**Figure 8:**
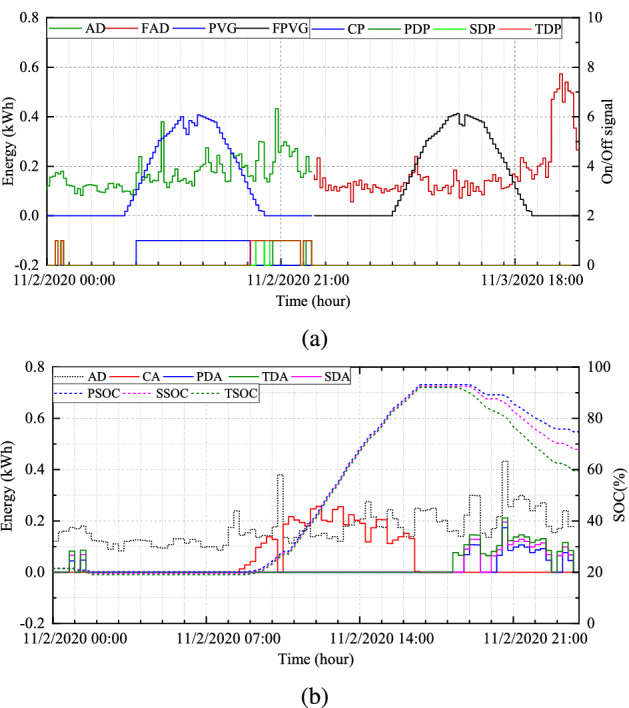
(**a**) Day-ahead results and scheduling periods and (**b**) charging, discharging, and SOC at normal condition for $H_{4}$.

We evaluated the proposed system performance by selecting another scenario ($S_{4}$) where the sum of day and day-ahead demand and PV generation are approximately equal. The function of the proposed system in the scenario is to fully discharge the BESS under the discharging allowance. Figure 8a demonstrates the scheduling plan for the three control modes by considering the day-ahead generation and demand. The discharge scheduling time for BESS in TCM is longer than discharging allowance in SCM and PCM.

**Figure 9:**
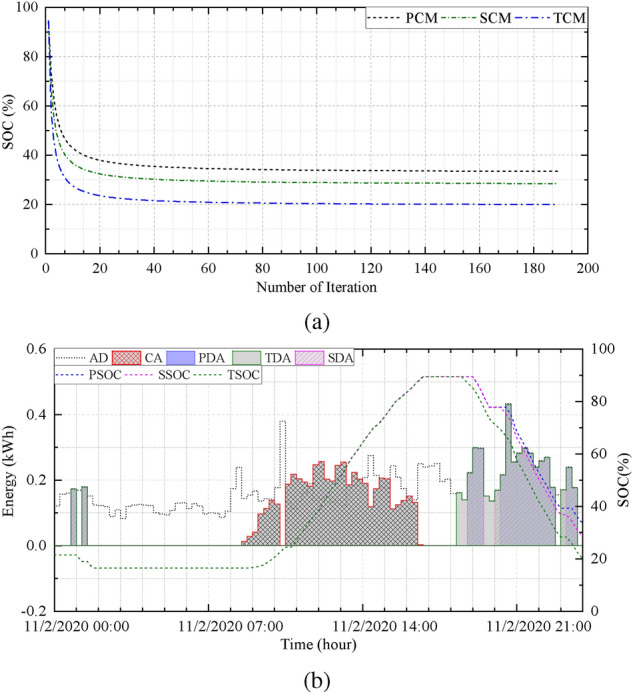
(**a**) Convergence curve, and (**b**) charging, discharging, and SOC at optimal condition for $H_{4}$.

The simulation results of $H_4$ under normal conditions are depicted in Fig.8b, where the SoCs of the BESS in TCM, SCM, and PCM at the ending period are $58.41\%$, $66.99\%$, and $74.11\%$. Under these conditions, the BESS cannot store excessive generated PV energy because of the higher SoC. Consequently, the system must be designed so that all constraints and dependencies are accomplished. As depicted in Fig.9b, the SoCs in TCM, SCM, and PCM are $33.41\%$, $28.46\%$, and $19.97\%$, which are adequate for improving the system performance in terms of reducing cost and expanding the usages of RES.

**Figure 10:**
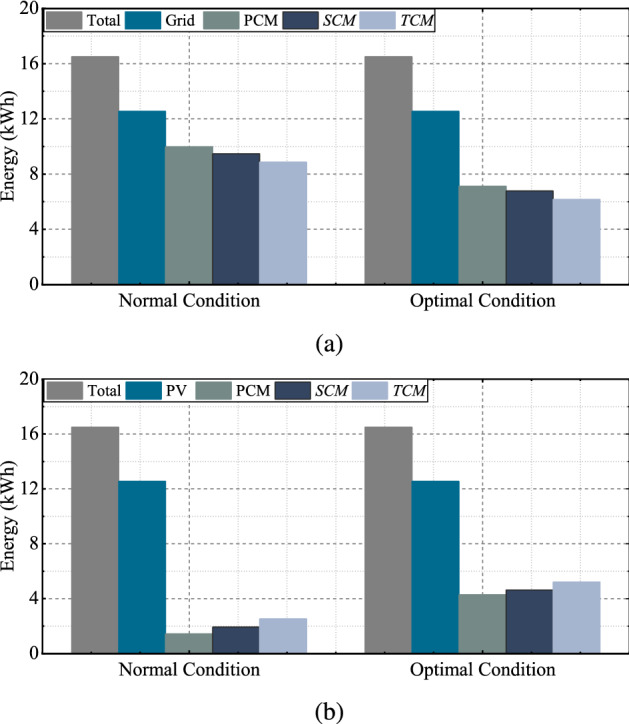
(**a**) Grid energy supply and (**b**) BESS energy supply at normal and optimal condition for $H_{4}$.

**Figure 11:**
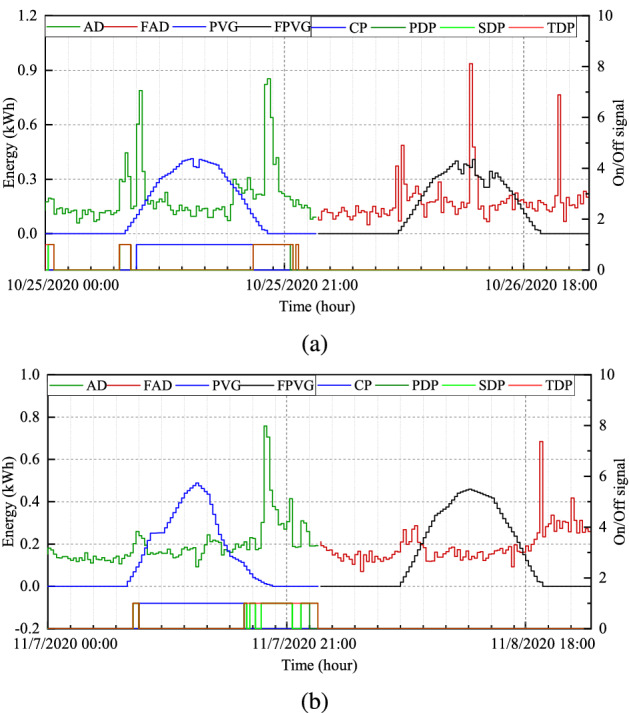
Day-ahead generation and consumption profile and scheduling periods for (**a**) $H_{2}$ (**b**) $H_{3}$.

The convergence graph and simulation results under optimal conditions are depicted in Fig. 9, and the volume of energy supplied by the grid and BESS is depicted in Fig. 10. Figure 11a,b illustrate the scheduling plan and its day-ahead demand and generation for $H_{2}$ and $H_{3}$ regarding scenarios $S_{2}$ and $S_{3}$. The comparative analysis of providing BESS energy during a single day in different control modes for each household is summarized in Table 5. Table 6 describes the electricity cost of a single day in different optimization modes for both cases [the per-unit cost of the electricity is calculated in the South Korean won ()]. In Table 7, the performance of the proposed scheme is described in terms of the percentage of cost minimization of a single day.

**Table 5:** Daily electricity usages [kWh] in different cases.

	Normal				Optimization			
	$H_{1}$	$H_{2}$	$H_{3}$	$H_{4}$	$H_{1}$	$H_{2}$	$H_{3}$	$H_{4}$
PDA	1.46	2.38	2.33	1.43	4.22	6.49	6.48	4.27
SDA	1.89	3.00	3.03	1.92	4.38	6.87	7.06	4.62
TDA	2.34	3.45	3.96	2.52	4.79	7.05	7.78	5.22
PGS	6.76	10.28	11.54	9.97	4.00	7.38	7.39	7.12
SGS	6.32	9.66	10.84	9.47	3.84	5.79	6.81	6.77
TGS	5.88	9.21	9.91	8.87	3.43	5.61	6.09	6.18

**Table 6:** Daily electricity cost [] in control mode.

Mode	Normal			Optimization		
	PCM	SCM	TCM	PCM	SCM	TCM
$H_{1}$	392.94	363.73	335.82	191.47	184.91	162.31
$H_{2}$	577.13	533.68	504.97	292.40	262.18	254.56
$H_{3}$	597.04	552.51	482.22	284.53	260.54	200.45
$H_{4}$	439.93	405.97	361.17	233.34	212.36	164.82

**Table 7:** Percentage of reduction of cost in a single day.

Mode	Normal			Optimization		
	PCM (%)	SCM (%)	TCM (%)	PCM (%)	SCM (%)	TCM (%)
$H_{1}$	8.85	11.24	13.51	25.28	25.81	27.66
$H_{2}$	11.90	14.98	17.01	32.08	34.22	34.76
$H_{3}$	12.03	15.03	19.77	33.10	34.72	38.77
$H_{4}$	7.86	10.38	12.71	23.20	24.76	28.29

We also analyzed the payback periods of the proposed system based on the scenarios. We investigated two types of houses to determine payback periods: a conventional house with no PV-BESS installed and a smart house with PV-BESS installed. We calculated the PV and BESS installation costs based on market pricing and used the approximation cost to determine the cost of the proposed control and management system. The overall cost of the proposed control and management system is added to the cost of installing PV-BESS in a conventional home, while the daily energy cost is calculated using a flat-rate tariff. In contrast , only the cost of the proposed control and management system is considered for a smart house, while the daily energy cost is calculated using a dynamic tariff.

Tables 8 and 9 represent the conventional and smart house’s daily savings and payback periods. The payback period and daily cost savings of a conventional house in the normal mode are greater than even a smart house because the conventional house uses PV-BESS and the proposed scheme simultaneously. Consequently, nearly the entire daily electricity bill can be saved. In contrast, the smart house is already saving money because of the PV-BESS installation and dynamic pricing. Implementing the proposed scheme will further reduce the costs, increasing daily cost savings . Furthermore, because of the lower installation cost of the proposed control and management technology, the smart house’s payback periods are shorter than traditional houses in optimization mode. The following explains the total cost analysis and payback times for the three control modes.

**Table 8:** Payback period analysis of the proposed system with the conventional house.

House number	Cost []			Normal mode						Optimization mode					
	PV-BESS installation	Control system installation	Per day(TOU)	Cost saving per day []			Payback period [year]			Cost saving per day []			Payback period [year]		
				PCM	SCM	TCM	PCM	SCM	TCM	PCM	SCM	TCM	PCM	SCM	TCM
$H_{1}$	4748176.00	457810.00	3005.44	2612.50	2641.71	2669.62	5.46	5.25	5.20	2813.97	2820.53	2843.13	5.07	5.06	5.02
$H_{2}$	5585642.00	457810.00	3783.60	3206.47	3249.92	3278.63	5.16	4.98	4.93	3491.20	3521.42	3529.04	4.74	4.70	4.69
$H_{3}$	5585642.00	457810.00	3777.80	3180.76	3225.29	3295.58	5.21	5.01	4.91	3493.27	3517.26	3577.35	4.74	4.71	4.63
$H_{4}$	4748176.00	457810.00	3301.60	2861.67	2895.63	2940.43	4.98	4.79	4.72	3068.26	3089.24	3136.78	4.65	4.62	4.55

**Table 9:** Payback period analysis of the proposed system with the PV-BESS integrated house.

House number	Cost []			Normal mode						Optimization mode					
	Control system installation	Per day(TOU)	With PV-ESS	Cost saving per day []			Payback period [year]			Cost saving per day []			Payback period [year]		
				PCM	SCM	TCM	PCM	SCM	TCM	PCM	SCM	TCM	PCM	SCM	TCM
$H_{1}$	457810.00	1226.59	501.55	108.61	137.83	165.74	11.55	9.10	7.57	310.08	316.64	339.24	4.05	3.96	3.70
$H_{2}$	457810.00	1410.72	744.97	167.84	211.29	240.01	7.47	5.94	5.23	452.57	482.79	490.42	2.77	2.60	2.56
$H_{3}$	457810.00	1483.18	775.51	178.46	223.00	293.28	7.03	5.62	4.28	490.98	514.96	575.06	2.55	2.44	2.18
$H_{4}$	457810.00	1346.46	545.72	105.78	139.74	184.55	11.86	8.98	6.80	312.37	333.36	380.89	4.02	3.76	3.29

### PCM

In PCM optimization mode, the simulation results for the scheduling of the BESS demonstrate a shorter period than in other control modes. The constraints are described in the Eqs. (43) and (45), and the potential supplied energy of the BESS is described by Eqs. (44) and (46). The discharging amount of the BESS is simple and finite and is not dependent on any other factors. From Table 6, the percentage of maximum cost reduction in a single day belongs to $H_{3}$: approximately $12.03\%$ under normal conditions and $33.10\%$ under optimal conditions. The cost reduction difference between normal and optimal condition for $H_{1}$, $H_{2}$, $H_{3}$, and $H_{4}$ are $16.42\%$, $20.18\%$, $21.07\%$, and $15.34\%$.

Furthermore, the higher percentages of daily electricity cost reduction lead the shorter payback periods for the household. While the home is considered a conventional house, the shorter payback periods correspond to $H_4$, which is 4 years, 11 months, and 22 days (approximately) under normal conditions and 4 years, 7 months, and 24 days (approximately) under optimal conditions. Furthermore, when the house is deemed a smart house, $H_3$ has the shortest payback times, which are 7 years and 10 days (approximately) under normal conditions and 2 years, 6 months, and 18 days (approximately) under optimal conditions.

### SCM

In this case, the constraints for day-ahead power generation are included, and the objective function is modified by the discharge factor for PV generation. The constraints are explained in Eqs. (47), (48), (49), (52), (53), and (54). The objective function is calculated in Eqs. (51) and (55). The results significantly improved compared with the PCM. The maximum and minimum percentages of the cost reduction are $15.03\%$ and $10.38\%$ under normal conditions and $34.72\%$ and $24.76\%$ under optimal conditions.

A conventional house requires a minimum of 4 years, 9 months, and 14 days (approximately), and a maximum of 5 years and 3 months (approximately) to compensate for the installation cost when their energy demand and PV generation are the same as $H_{4}$ and $H_{1}$ under normal conditions. In contrast, when the energy consumption and PV generation are the same as $H_3$ and $H_1$ under optimal conditions, a smart house requires a minimum of 2 years, 5 months, and 8 days (approximately), and a maximum of 3 years, 11 months, and 16 days (approximately) to recover the installation cost. Consequently, under optimal conditions, smart homes gain a significant improvement in payback time because of lower installation costs.

### TCM

The highest optimization scheme of the proposed model is governed by multiple factors and a maximum number of constraints. The dynamic tariff constraint and tertiary discharging factor were added for optimal discharging. Additional constraints in Eqs. (56) and (59) are provided for the control mode. The percentage of minimization of electricity cost is the highest compared with any other model in the system. In this control mode, the highest cost optimization is obtained in $H_{3}$ under optimal conditions, and the difference between the two conditions is $19.77\%$. Similarly, $H_{1}$ and $H_{4}$ achieve the lowest optimization of about $14.15\%$ and normal optimization of about $15.58\%$, as anticipated.

Under the TCM, the proposed system provides much shorter payback periods for the households both conventional and smart households. Table 8 reveals that any conventional house with the same energy use and PV generation as the households in this study may recoup its installation and operational costs in four to five years (approximately). Similarly, any smart house can recover the operational cost of the proposed control and management scheme within two to four years (approximately).

## Conclusion

This paper proposed an energy management model in the home environment considering the operational constraints of appliances, BESS, and dynamic tariff systems. A PV-BESS controller needs reliable forecasting and robust scheduling algorithms to maximize renewable power utilization and minimize daily electricity costs. Introducing artificial intelligence (AI)-based operational dependencies with the optimization algorithm reduces the gap between generation and consumption, enhancing the robustness and performance of scheduling and optimization results.

Numerical analysis and simulation were conducted on the different scenarios, and control mode schemes demonstrated the effectiveness and robustness of the system. The applied day-ahead load forecasting model achieved an optimal performance: $13.69\%$, 0.017 kW, and 0.029 kW for the MAPE, MSE, and MAE. Similarly, the model for day-ahead PV generation prediction yielded optimal results: $16.421\%$, 0.0001 kW, and 0.0061 kW for the MAPE, MSE, and MAE.

Moreover, the maximum improvement is observed in the case of TCM under optimal conditions, while the proposed system is capable of reducing the maximum to approximately $38.77\%$ (under $S_{3}$) and a minimum of $27.66\%$ (under $S_{1}$) for daily electricity cost. Therefore, it achieves the highest and lowest utilization of BESS energy of approximately $41.19\%$ (under $S_{3}$) and $31.59\%$ (under $S_{1}$) of daily energy demand, resulting in significant performance under the complexity and constraints inherent in HEMS. In the future, a study will be conducted to improve on the present formulation by including congestion management, which will incorporate PV, wind, and numerous BESSs. Another target of the future research is to implement the proposed system at the hardware level.

## Data Availability

The data that support the findings of this study are available from Information Technology Research Center (ITRC) but restrictions apply to the availability of these data, which were used under license for the current study, and so are not publicly available. Data are however available from the authors upon reasonable request and with permission of ITRC.
